# Digestible Food

**Published:** 1896-09

**Authors:** 


### Digestible Food.

One of the biggest mistakes about food which people make, says the London *Hospital*, is to forget that the true value of food to anybody is in the digestibility. Half a pound of cheese is vastly more nourishing, as regards its mere components, than half a pound of beef, but while the beef will be easily digested, and thus be of vast service to us, the cheese is put out of court altogether for ordinary folks by reason of its indigestibility.—*N. Y. Med. Times.*

According to recent experiments described in *The Medical Record* each pint of air breathed by an adult contains about 15,000 microbes. In some places the number is as high as a million, but the average city number is about as stated. This microbe-laden air is taken into the air-passages, and when it is thrown out it is quite sterile. The air has further been found to be sterile in the naso-pharyngeal cavity. The inference is that the nose is a most powerful microbe-destroyer, and this fact shows also how important it is to draw the air through the nasal passages.

